# Adherence to Compression Garments in Lymphedema Patients: A Cross-Sectional Study

**DOI:** 10.3390/medicina61040685

**Published:** 2025-04-08

**Authors:** María Elena Medina Rodríguez, Raúl Socorro Suárez, Manuel Albornoz Cabello, Carolina Aranda Rodríguez, Pino Delia Domínguez Trujillo, Victoria Peña Curbelo

**Affiliations:** 1 Department of Medical-Surgical Sciences, University of Las Palmas de Gran Canaria, Campus de San Cristóbal, 35016 Las Palmas, Spain; 2 Physiotherapy Unit, Hospital Perpetuo Socorro Hospital, C/León y Castillo, 35007 Las Palmas, Spain; 3 Department of Physiotherapy, University of Seville, C/Avicena s/n, 41009 Seville, Spain; 4 Rehabilitation Unit, General Hospital of Gran Canaria Dr. Negrín, Barranco de la Ballena s/n, 35010 Las Palmas, Spain; 5 PhD Programme in Medicine and Biomedical Sciences, Doctoral School, Universitat de Vic—Central University of Catalonia (UVic-UCC), Sagrada Familia, 08500 Vic, Spain

**Keywords:** lymphedema, compression garments, adherence, compliance

## Abstract

*Background/Objectives*: This study evaluated adherence to compression garments, their role in edema stabilization, and factors influencing patient compliance. *Materials and Methods*: This observational, descriptive, cross-sectional study evaluated adherence to compression garments in 92 patients with lymphedema treated at the University Hospital of Gran Canaria Dr. Negrín. In addition, sociodemographic, clinical, and design-related factors influencing adherence were analyzed. The evaluation was conducted via telephone questionnaires and a review of medical records. *Results*: The study revealed low adherence to compression garments in patients with lymphedema. Adherence was related to the garments’ etiology, severity, and perceived comfort. *Conclusions*: The findings highlight the need to improve education strategies and personalize treatment recommendations to enhance adherence.

## 1. Introduction

Lymphedema is a chronic condition marked by the accumulation of lymphatic fluid in tissues, causing swelling due to lymphatic system dysfunction [1]. It can be congenital (primary lymphedema) or acquired (secondary lymphedema), with the latter often resulting from surgeries, radiotherapy, infections, or trauma, particularly after breast cancer treatment in developed countries [2,3]. There is no definitive cure [4,5], but Complex Decongestive Therapy (CDT) is the standard treatment [6,7]. CDT involves manual lymphatic drainage, compression garments, skin care, and exercise, divided into an intensive phase for volume reduction and a maintenance phase focused on sustaining results through self-care practices like compression garments and exercise [4,7,8,9].

Compression garments are vital in maintaining edema stabilization by improving lymphatic drainage [10], reducing fluid leakage, lowering infection risk, and enhancing patients’ quality of life [11,12]. Custom-made garments are preferred for severe cases due to better adaptability, while standard garments are more cost-effective [13,14,15]. Proper prescription and patient factors, such as limb morphology, habits, and financial support, influence garment selection [6,10,13,14,16,17,18].

Adherence to lifelong self-management is crucial for controlling lymphedema, preventing treatment failure, and improving quality of life [5,7,19,20]. However, adherence rates (28–69%) decline over time due to discomfort, time requirements, and interference with daily activities [12,13,16,18,21,22]. This study evaluated adherence to compression garments, their role in edema stabilization, and factors influencing patient compliance.

## 2. Materials and Methods

### 2.1. Study Design and Population

This observational, descriptive, cross-sectional study was conducted at the University Hospital of Gran Canaria, Doctor Negrín (H.U.G.C. Dr. Negrín), Las Palmas, Spain, between September 2022 and May 2023. A cohort of 105 adult subjects with a medical diagnosis of lymphedema who had been treated with CDT at the Lymphedema Unit of the Rehabilitation Service of this hospital between 2019 and 2021 was recruited. The patients were identified through a review of electronic medical records to determine their eligibility for inclusion in the study. The Biomedical Research Ethics Committee (CEIB) of H.U.G.C. Dr. Negrín approved this study protocol (CEIC Negrín Code 2022-436-1).

**Figure 1:**
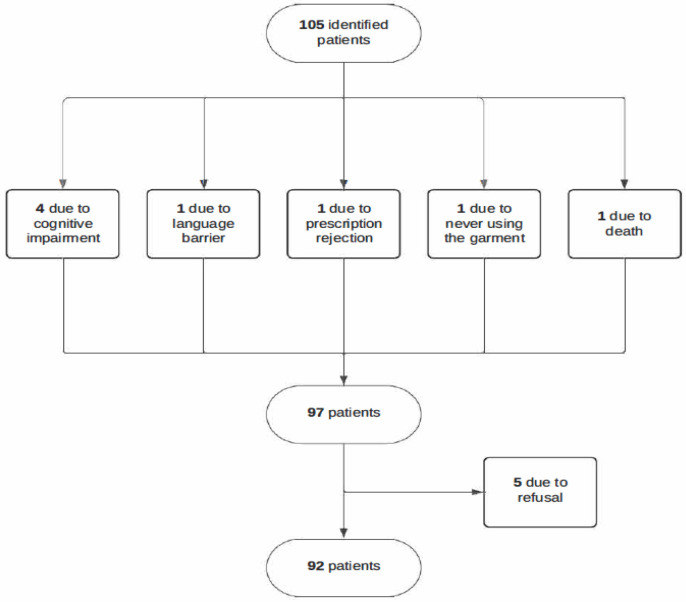
Recruitment process for the study.

The exclusion criteria were (1) being under 18 years of age, (2) not completing the intensive CDT phase at the unit, (3) not obtaining the prescribed compression garment after the intensive phase of physical treatment, (4) being recorded as deceased in the institution’s database, (5) not understanding the Spanish language, (6) having a disease or cognitive impairment that prevented them from responding, or (7) not providing informed consent before the study. After applying the eligibility criteria, the study population consisted of 92 subjects, 59 of whom had unilateral upper limb lymphedema, and the remaining 33 had unilateral lower limb lymphedema (Figure 1). All the participants freely provided their informed consent before participating in the study.

### 2.2. Variables of Interest

The primary dependent variable in this study was adherence to the use of compression garments during the maintenance phase of lymphedema, which, based on bibliographic evidence recommendations, was defined as wearing the garment for more than 12 h per day during the daytime.

In addition, other variables of interest were collected: sociodemographic information, comorbidities, lifestyle habits, etiology and characteristics of lymphedema, and factors associated with the design and use of compression garments. To reduce the subjectivity of self-assessment of edema severity, three possible outcomes were established based on its visibility [23]: mild (only perceived by the patient), moderate (noticed by someone close to the patient), or severe (noticed by anyone).

Data collection was conducted through two main sources: the patient’s medical records, available in the hospital’s database, and a self-designed questionnaire (Supplementary Material File S1). Since no validated questionnaires were found in the literature at the time, a new one was developed with the guidance of expert researchers with training and experience in the medical diagnosis and clinical treatment of lymphedema. The questionnaire was administered via telephone. The questionnaire’s suitability was tested in a pilot trial.

### 2.3. Statistical Analysis

The adherence to compression garments was evaluated via the R statistical platform (version 3.4.1).

For the univariate descriptive analysis, qualitative variables were summarized via absolute and relative frequencies, whereas quantitative variables were presented via medians and the first and third quartiles (Q1–Q3). Adherence was defined as wearing the compression garment for over 12 h daily.

Exploratory data analysis was conducted to identify significant variables through univariate logistic regression. Variables with a *p*-value < 0.25 were selected for inclusion in the multivariate regression models. These variables were adjusted using a logistic regression model with forward and backward selection approaches. The final model was selected based on the Akaike Information Criterion (AIC), choosing the lowest AIC value.

The multicollinearity between predictor variables was assessed via the Variance Inflation Factor (VIF), where all values were less than 5, indicating no serious collinearity issues. The Hosmer–Lemeshow test was also conducted to evaluate the model’s goodness of fit.

Finally, associations are presented as odds ratios (ORs) with their respective 95% confidence intervals. The level of statistical significance considered for all analyses was *p* < 0.05.

## 3. Results

### 3.1. Patient Characteristics

In total, 92 of 105 selected subjects completed the questionnaire (response rate of 87.62%). The sample predominantly consisted of women (91.3%), and the median age was 65 years (range: 54–73). Among the participants who reported their education level, 45.7% had primary education, 25% had secondary education, and 25% had a university degree. Concerning employment status, 26.1% were employed, 26.1% were unemployed, 42.4% were retired, and 5.4% were on temporary disability leave. Among the employed population, 68.5% were homemakers.

The age (*p*-value 0.22), sex (*p*-value 0.51), educational level (*p*-value 0.79) or the employment status (*p*-value 0.76) were not associated with greater adherence to compression garments.

### 3.2. Clinical Status of Lymphedema Patients and Follow-Up

Regarding the etiology of lymphedema, 16.3% (*n* = 15) of the patients had the primary etiology; among these patients, 66.7% (*n* = 10) demonstrated good adherence. In contrast, 66.2% of the patients with secondary lymphedema (*n* = 77) did not show good adherence. Therefore, primary lymphedema etiology appears to be associated with better adherence to compression garments (*p*-value = 0.02). Data analysis revealed that the location of the affected anatomical region (upper or lower limb) influences adherence (*p*-value = 0.03). Patients with lower limb lymphedema showed greater adherence to compression garment use than those with upper limb lymphedema. Although edema severity was not directly related to adherence (*p*-value = 0.24), the data analysis indicated that patients with more severe edema exhibited better adherence than moderate or mild edema (Table 1).

Concerning follow-up, 85.9% (*n* = 79) of patients attended regular medical check-ups, and most patients (68.5%, *n* = 63) attended check-ups every 5–6 months. The frequency of medical check-ups and attendance at follow-up visits were not associated with adherence to compression garments (*p*-value = 0.96).

### 3.3. Types of Garments and Usage Habits

Regarding the type of garment, 95.7% (*n* = 88) of the garments were custom-made. Custom-made garments showed good adherence in 39.8% (*n* = 35) of the cases. Standard garments had a lower proportion of good adherence (25.0%, *n* = 1). Most patients (88%) considered putting on compression garments easy, and 81.5% stated that they were independent when putting them on. Additionally, 29 participants (31.52%) acquired a device to facilitate garment placement. Adherence, defined as wearing the compression garment continuously for more than 12 h, was 39.13%. Regarding the frequency of use, 78.26% (*n* = 72) of the participants used garments 6–7 days per week. Furthermore, the use of garments during weekends, household chores, work, and leisure activities was typical, with a high proportion of use in all contexts (>90%). Most patients (78.26%, *n* = 72) put on their garments immediately upon waking. Among those who perceived the garments as comfortable (79.35%, *n* = 73), 45.2% (*n* = 33) exhibited good adherence. The reasons for not renewing the garments within the recommended timeframe (every six months) included the perception that the garment was in good condition (28.26%), missing follow-up appointments (19%), financial issues (2%), and improvement in symptoms with the perception of no longer needing the garment (2%).

### 3.4. Adherence to Compression Systems

Adherence, defined as the continuous use of compression garments for more than 12 h per day, was 39.13%. In the initial model, the variables “Affected Limb”, “Etiology”, “Severity”, “Number of Garments”, “Perception”, and “Age” were selected based on a *p*-value of less than 0.25 in the univariate analysis. Both forward and backward variable selection procedures were performed, and the final model was chosen via backward selection, as it presented a lower AIC value (109.313 compared with 111.8739).

According to this model, patients with primary lymphedema were more likely to adhere to compression garment use than those with secondary lymphedema (OR: 8.86; 95% CI: 1.43, 54.93; *p* = 0.01). Additionally, patients with severe edema were 4.57 times more likely to adhere to treatment than those with moderate edema (OR: 4.57; 95% CI: 1.22, 17.04; *p* = 0.035). The perception of comfort while wearing the garments was strongly associated with greater adherence, as those who found the garment comfortable had an adjusted OR of 13.51 (95% CI: 2.35, 77.59; *p* < 0.001) compared with those who found it uncomfortable (Table 2).

## 4. Discussion

The primary objective of this study was to assess adherence to compression systems (sleeves and stockings) in patients with lymphedema. Adherence was defined as the proportion of the actual sleeve and stocking use frequency relative to the prescribed frequency [24]. Our data analysis revealed that 39.13% of the 92 participants adhered to the prescribed recommendation of wearing compression garments for more than 12 h daily. Similarly, a 39% adherence rate has been reported [25]. Other studies have reported rates ranging from 51.7% [13] to 78.7% [26] for upper limb lymphedema.

In terms of etiology, patients with primary lymphedema are almost nine times more likely to adhere to the recommended use of compression garments than are those with secondary lymphedema (OR: 8.86; 95% CI: 1.43, 54.93; *p* = 0.01). The perception of the importance of treatment may be higher in patients with primary lymphedema than in those with secondary lymphedema, where lymphedema may be considered just another complication of the underlying condition. Sleeves are more difficult to conceal than stockings, and the former may interfere with the functionality of the upper limb in daily activities [25].

Univariate analysis showed no significant relationship between edema severity and treatment adherence (*p*-value = 0.24). However, multivariate analysis indicated that patients with severe edema were more likely to adhere to treatment (*p*-value = 0.035), likely due to experiencing more intense symptoms that motivate adherence.

Maximizing adherence to compression garments requires patients to understand their purpose and proper use [27], along with clear recommendations [26]. The literature lacks consensus on usage guidelines; some emphasize activity type or limb position, while others focus on wearing time, ranging from 6 to over 20 h daily [16,28]. Authors generally advise daily wear during waking hours [6,9,12,13,16], especially for moderate or severe edema [26], and during exercise [5,13,28,29,30] or when the limb is in a disadvantaged position [10].

The effectiveness of compression garments has been linked to both the frequency of use and their replacement [31]. Frequent washing, exposure to sunlight and body oils, and the frequent putting on and taking off of compression sleeves or stockings can affect their compression [11,13,32] and, consequently, their effectiveness in stabilizing edema. In our study, garments were renewed in most cases, regardless of the location and severity of the edema, every 12 months or more. Medical check-ups in our study were conducted every 5–6 months in 68.5% of the patients. According to most authors and working groups, garments should be replaced when a loss of compression is perceived (increased elasticity) or based on the usage period, which is recommended to be every six months or even sooner [11,13,15,27,33,34,35]. The patient’s physical activity should also be considered when determining the replacement frequency; younger or more active patients may need to replace their garments more [31]. Patients are recommended to have two garments simultaneously and alternate their daily use. In this regard, volumetric control during periodic check-ups is crucial to verify the garment’s effectiveness.

In addition to the limb’s morphology, factors such as edema severity, comfort, and cost play crucial roles in adherence to compression garments [10,11,15,17,22,35]. The patient’s economic situation significantly influences the choice and renewal of these garments [13,16,17,25,36]. Standard sleeves are generally less expensive than custom-made options, and financial support from health systems is vital. While some patients receive partial or complete coverage, many face significant copayments [17,36,37]. In our system, patients must contribute EUR 30, which often does not cover the total cost. Although garment renewal is allowed every six months, our study found that they are often renewed only annually. Limited government support in some countries may further hinder access to these garments [35]. This financial barrier can negatively impact adherence, particularly in long-term treatment, where replacement frequency and garment quality are essential to maintaining therapeutic efficacy. Therefore, increased public health support could be a key strategy to improve overall adherence rates.

This study has limitations, such as relying on patient self-reports for adherence, which may introduce bias. In particular, key variables such as the duration of garment use, the perceived fit of the compression system, and the severity of edema were based entirely on patients’ subjective perceptions. This reliance may lead to recall or perception bias, potentially affecting the accuracy of the results. Although self-reported data are commonly used in clinical contexts due to their feasibility, future research should focus on developing and validating objective tools that allow for a more reliable assessment of adherence to compression therapy. Another important limitation is the relatively small sample size (92 patients), which may limit the generalizability of the findings and preclude more detailed subgroup analyses, particularly regarding patients with primary lymphedema and those with lower limb involvement, who were underrepresented.

Despite its limitations, this study offers valuable insight into the barriers and behaviors associated with adherence to compression garments in a specific patient population. By identifying key factors that hinder or facilitate adherence, our findings may help inform targeted interventions aimed at increasing compliance with recommended usage guidelines. Ultimately, adopting these strategies could improve clinical outcomes and quality of life for patients with lymphedema.

Future research should aim to expand upon these findings by including larger and more diverse populations, allowing for stratified analysis by lymphedema type, limb involvement, and demographic factors. Additional studies should also explore the psychological, social, and economic barriers to long-term compression therapy adherence and the potential impact of patient education and health system support. Moreover, future research should utilize objective measurements of edema volume and consider adapting recommended usage times based on edema severity and patients’ daily routines to enhance treatment effectiveness. These directions would contribute to designing more effective, equitable, and patient-centered interventions to improve treatment outcomes.

## 5. Conclusions

In conclusion, improving adherence to compression garments in patients with lymphedema is essential to prevent disease progression and maintain the benefits achieved during the intensive phase of treatment. The results show that only 39.13% of patients adhered to the recommendation of wearing garments for more than 12 h daily, highlighting the need to improve adherence in this population. Greater adherence to compression garments was associated with primary etiology, greater edema severity, and the perception of comfort while the garment was used.

## Figures and Tables

**Table 1:** Summary of variables related to lymphedema characteristics and follow-up.

			Adherence		
Variable	Categories	Total	Yes *n* = 36	No *n* = 56	*p*-Value ^a^
Etiology	Primary	15 (16.3%)	10 (66.7%)	5 (33.3%)	0.02
	Secondary	77 (83.7%)	26 (33.8%)	51 (66.2%)	
Affected Limb	Lower limb	33 (35.9%)	18 (54.5%)	15 (45.5%)	0.03
	Upper limb	59 (64.1%)	18 (30.5%)	41 (69.5%)	
Edema Severity	Mild	17 (18.5%)	7 (41.2%)	10 (58.8%)	0.24
	Moderate	57(62.0%)	18 (31.6%)	39 (68.4%)	
	Severe	18(19.6%)	11 (61.1%)	7 (38.9%)	
Medical Check-Ups	Yes	79(85.9%)	31 (39.2%)	48 (60.8%)	0.96
	No	13(14.1%)	5 (38.5%)	8 (61.5%)	
Check-Up Frequency	Every 3–4 months	3 (3.3%)	1 (33.3%)	2 (66.7%)	0.96
	Every 5–6 months	63 (68.5%)	22 (34.9%)	41 (65.1%)	
	Every year	26 (28.3%)	13 (50.0%)	13 (50.0%)	

^a^: Univariate logistic regression.

**Table 2:** Multivariate analysis of adherence to compression garments.

	OR (IC95%)	AOR (IC95%)	*p*-Value
Affected Limb			0.146
Upper limb			
Lower limb	2.73 (1.13, 6.6)	2.36 (0.74, 7.52)	
Etiology			0.01
Secondary			
Primary	3.92 (1.21, 12.68)	8.86 (1.43, 54.93)	
Severity Level			0.035
Moderate			
Severe	3.4 (1.13, 10.23)	4.57 (1.22, 17.04)	
Mild	1.52 (0.5, 4.63)	2.74 (0.73, 10.32)	
Perception of Use			<0.001
Uncomfortable			
Comfortable	4.4 (1.18, 16.41)	13.51 (2.35, 77.59)	

OR: odds ratio. AOR: adjusted odds ratio.

## Data Availability

Dataset available on request from the authors.

## Supplementary Materials

The following supporting information can be downloaded at: https://www.mdpi.com/article/10.3390/medicina61040685/s1, File S1: Questionnaire administered to patients.

## Abbreviations

CDT	Complex Decongestive Therapy
H.U.G.C.	University Hospital of Gran Canaria
CEIB	Biomedical Research Ethics Committee
ORs	odds ratios
