# Supplementary material for: Adherence to Compression Garments in Lymphedema Patients: A Cross-Sectional Study
Source: Medicina (Kaunas). 2025 Apr 8;61(4):685. doi: 10.3390/medicina61040685 (PMC12028548; doi:10.3390/medicina61040685)
Supplement: Supplementary file 1 [file medicina-61-00685-s001.zip › medicina-3527205-supplementary.pdf]

# EVALUATION OF ADHERENCE TO LYMPHOEDEMA CONTAINMENT SYSTEMS IN PATIENTS AT THE GENERAL HOSPITAL OF GRAN CANARIA DR. NEGRÍN BETWEEN 2019-2021.

* Indicates that the question is mandatory

1. DISCHARGE DATE WITH PLEDGE *

____

Example: 7 January 2019

2. REGISTRATION NUMBER *

____

3. SEX *

Mark only one oval.

- ◯ MALE
- ◯ FEMALE
- ◯ Other: _______________________________

4. DATE OF BIRTH *

____

Example: 7 January 2019

5. DOMINANT SIDE *

Mark only one oval.

- RIGHT
- LEFT
- BOTH

ASSOCIATED WITH CLINICAL SITUATION

6. CONCOMITANT PATHOLOGIES *

Mark only one oval.

- YES
- NO

7. WHICH?

____

CHARACTERISTICS OF LYMPHOEDEMA

8. AFFECT SIDE *

Mark only one oval.

- RIGHT
- LEFT
- BOTH

9. AFFECTED MEMBER *

Mark only one oval.

- ( ) UPPER
- ( ) LOWER

10. REGION OF THE BODY AFFECTED *

- ( ) HAND
- ( ) FOREARM
- ( ) ARM
- ( ) WHOLE ARM
- ( ) FOOT
- ( ) LEG
- ( ) THIGH
- ( ) THE WHOLE LEG

11. AETIOLOGY OF LYMPHOEDEMA *

Mark only one oval.

- ( ) PRIMARY
- ( ) SECONDARY

12. TIME OF EVOLUTION OF THE OEDEMA *

____

13. LEVEL OF OEDEMA *

Mark only one oval.

- SLIGHT
- MODERATE
- SEVERE

14. GO FOR REGULAR MEDICAL CHECK-UPS

Mark only one oval.

- Yes
- No

15. IF , WHAT IS THE REASON?

____

16. FREQUENCY OF REVISIONS IN THE LAST YEAR

Mark only one oval.

- EVERY 1- 1 - 2 MONTHS
- EVERY 3-4 MONTHS
- EVERY 5-6 MONTHS
- EVERY YEAR

17. WHEN WAS THE LAST REVIEW? *

____

18. NUMBER OF TOTAL REVIEWS (since start of treatment) * NUMBER OF TOTAL REVIEWS (since start of treatment) * NUMBER OF TOTAL REVIEWS (from start of treatment)

FREQUENCY OF RENEWAL OF CONTAINMENT SYSTEMS

IF YOU HAVE EVER USED CONTAINMENT SYSTEMS

19. DATE OF THE LAST PLACEMENT OF THE PLEDGE *

Mark only one oval.

- 1-2 MONTHS AGO
- 3-4 MONTHS AGO
- 5-6 MONTHS AGO
- 6-12 MONTHS AGO
- MORE THAN 12 MONTH AGO

20. HOW MANY GARMENTS HAVE YOU WORN SINCE THE START OF TREATMENT? *

Mark only one oval.

- 1
- 2
- 3
- 4
- 5
- 6

21. IN CASE OF NON-RENEWAL, WHAT WAS THE REASON?

Mark only one oval.

- ECONOMIC
- STABLE AND GOOD CONDITION OF THE GARMENT
- DOES NOT ATTEND MEDICAL CHECK-UPS
- PALPABLE IMPROVEMENT OF THE AFFECTED LIMB
- ANOTHER ONE

22. IF "OTHER", WHICH ONE?

____

CHARACTERISTICS OF CONTAINMENT SYSTEMS

23. WHAT IS THE TYPE OF GARMENT DESIGN YOU CURRENTLY WEAR? *

Mark only one oval.

- STANDARD
- MADE MEASURE

24. IS THE GARMENT A ONE-PIECE OR TWO-PIECE? *

Mark only one oval.

- ONE PIECE
- TWO PIECES

25. GARMENT SIZE *

Mark only one oval.

- GLOVE
- GLOVE AND FOREARM
- GLOVE, FOREARM AND ARM
- KNEE-HIGH SOCKS
- STOCKING TO
- STOCKING WITH WAIST SUPPORT PANTYHOSE WITH
- PANTY GIRDLE AND EXTENSION
- PANTYHOSE

26. GARMENT COMPREHENSION CLASS *

Mark only one oval.

- CLASS I
- CLASS II
- CLASS III
- CLASS IV

27. HAS POSITIONING DEVICES *

Mark only one oval.

- YES
- NO

RELATED TO THE WAY COMPRESSIVE GARMENTS ARE USED

28. IT IS INDEPENDENT OF THE POSITIONING OF THE PART *.

Mark only one oval.

- YES
- NO

29. EASY FOR YOU PLACE *

Mark only one oval.

- YES
- NO

30. PERCEPTION WHEN WEARING THE GARMENT *

Mark only one oval.

COMFORTABLE ◯

UNCOMFORTABLE ◯

31. IF IT IS "UNCOMFORTABLE" WHAT IS THE REASON?

____

32. HOW LONG DO YOU USE THE GARMENT PER ? *

Mark only one oval.

◯ 2 HOURS
◯ 4 HOURS
◯ 8 HOURS
◯ 12 HOURS
◯ MORE THAN 12 HOURS

33. HOW MANY DAYS A WEEK DO YOU WEAR THE GARMENT? *

Mark only one oval.

◯ 1-2 DAYS
◯ 3-4 DAYS
◯ 5-6 DAYS
◯ 6-7 DAYS

34. DO YOU WEAR THE GARMENT ON ? *

Mark only one oval.

- YES
- NO

35. USE THE GARMENT OVERNIGHT *

Mark only one oval.

- YES
- NO

36. WHEN LIFTING, HOW LONG DOES IT TAKE TO PUT ON THE GARMENT? *

Mark only one oval.

- AS SOON AS I GET UP
- 2 HOURS AFTER GETTING UP
- 4 HOURS AFTER GETTING UP
- 6 HOURS AFTER WAKING UP
- 8 HOURS AFTER WAKING UP

37. DO YOU WEAR THE GARMENT DURING HOUSEHOLD CHORES? *

Mark only one oval.

- YES
- NO

38. DO YOU WEAR THE GARMENT DURING WORK DUTIES? *

Mark only one oval.

YES ◯

NO ◯

39. DO YOU WEAR THE GARMENT DURING LEISURE ACTIVITIES? *

Mark only one oval.

YES ◯

NO ◯

40. HAS ANY INCONVENIENCE WHEN WEARING THE GARMENT *.

Mark only one oval.

◯ ALWAYS

◯ NEVER

◯ SOMETIMES

41. DESCRIBE THE DRAWBACK

Mark only one oval.

◯ DISCOMFORT

◯ UNSIGHTLY

◯ LONG TIME PUT

◯ OTHERS

42. IF "OTHER", WHICH ONE?

43. DESPITE WEARING THE GARMENT. *
HAS YOUR ARM OR LEG SWOLLEN AGAIN?

Mark only one oval.

- Yes
- No

ASSOCIATED WITH THE PATIENT'S HABITS

44. IS PHYSICALLY ACTIVE *

Mark only one oval.

- YES
- NO

45. INTENSITY OF PHYSICAL ACTIVITY

Mark only one oval.

- LOW INTENSITY
- MEDIUM INTENSITY
- HIGH INTENSITY

RELATED TO EDUCATIONAL BACKGROUND AND JOB PERFORMANCE

46. WHAT LEVEL OF EDUCATION DO YOU HAVE? *

Mark only one oval.

- WITHOUT STUDIES
- PRIMARY EDUCATION
- SECONDARY EDUCATION
- UNIVERSITY STUDIES

47. CURRENT EMPLOYMENT SITUATION *

Mark only one oval.

- ACTIVE
- TEMPORARY INCAPACITY TO WORK
- TOTAL INCAPACITY TO WORK
- UNEMPLOYED
- RETIREE
- PENSIONER

48. CURRENT PROFESSION

Mark only one oval.

- HOUSEWIFE
- HEALTH CARE
- ADMINISTRATION
- OTHERS
